# Design and development of an IoT-based trolley for weighing the patient in lying condition

**DOI:** 10.3389/fdgth.2024.1339184

**Published:** 2024-09-30

**Authors:** S. Meenatchi Sundaram, Jayendra R. Naik, Manikandan Natarajan, Aneesha Acharya K

**Affiliations:** ^1^ Department of Instrumentation and Control Engineering, Manipal Institute of Technology, Manipal Academy of Higher Education (MAHE), Manipal, India; ^2^ Department of Physiotherapy at Manipal College of Health Professions, Manipal Academy of Higher Education (MAHE), Manipal, India

**Keywords:** scales, patients, IoT, microcontroller, weighing machine, load cell, clinical

## Abstract

**Introduction:**

An immobile patient cannot be weighed on a stand-on weighing machine, i.e., a bathroom scale. They have to get weighed while lying, which is not easy. The main objective of this research is to design a medical apparatus that measures the patient's weight in a lying condition. To achieve this the apparatus is designed as a stretcher to carry the patient in and around the hospital.

**Methods:**

The stretcher has four load cells to measure the patient's weight; it can bear a weight of 500 kg and has a self-weight of 20 kg. A Microcontroller unit (MCU) is embedded into the apparatus to weigh the patient lying on it. The stretcher comprises the top frame, middle frame, and base frame. The top frame can be detached and mounted back to the middle frame; this will help the medical personnel shift the patients from a medical bed. The middle frame is a plate structure where the four load cells are mounted at the corners of the lower plate. The upper plate functions as a pressure plate on the load cell. The base plate has four heavy-duty wheels that can bear the load. The middle frame and base frame, together, form a single structure, giving mobility to the structure. A control panel is employed with reset, tare, and on-off buttons to control the embedded platform. The LCD panel on the side of the apparatus shows the weight when the patient is placed on top of the apparatus.

**Results and discussion:**

A prototype trolley equipped with a wireless data logging system was tested on 10 healthy participants. The device accurately measured weight within ±50 g across a scale range of 2–140 kg, with data captured every 30 s over a 5-min testing period. Wireless communication was successfully demonstrated over a 100-m range. The important add-on feature of this work is the apparatus is connected to the internet, transforming it into an IoT-based medical device.

## 1 Introduction

Body weight measurement is an important assessment in the medical field. Body weight is essential for various vital decisions to be made during the rehabilitation of a patient. Some of the uses of weight assessment include calculation of body mass index, prescribing and calculating ideal dosage of medicines, fluid administration, selecting suitable beds to prevent pressure sores, providing appropriate dose of anesthesia before surgery, and assessing response to therapy such as dialysis and diuretics in renal and cardiac patients (1). Thus, patient weight is essential for medication safety and infection management (2). Accurately documenting a patient's body weight is essential for nutrition screening purposes and for any subsequent measures that may be necessary during treatment, such as accurate drug dosage and management of fluid accumulation (edema) or loss [Institute for Safe Medication Practices Canada (3)]. When patients are admitted, precise body weight measurement is particularly crucial since specialized equipment, like pressure-relieving profiling beds, may be required (4). Weight-predicting equations that use measuring tape are the only tools available in most clinical setups for estimating weight in bedridden individuals or those who cannot stand (5). All these methods are complex and rough estimates, which cannot be used to make appropriate decisions for rehabilitation based on body weight. Thus, there is a need for a device that can measure an individual's body weight in a lying position. The drug dosage prescription depends on the weight of the patient. A study (6) was conducted on the accuracy of weight and height recordings of patients made by intensive care unit (ICU) staff. The author's approach is to find the error occurring due to the visual estimation of patient weight and height in the intensive care unit. They have concluded that the height estimation has a smaller percentage error, while estimation in weight has an overall around 10 percent error in actual weight. A study was conducted on the lack of recording of the patient's weight in hospitals (7). This study shows that patients’ weight measurement is recorded poorly in the hospitals. The most frequent barrier to weighing the patient was due to workflow interruption and heavy staff workload. Various tools are available to measure body weight, of which digital weighing machines and bathroom scales are the most common. These tools require the individual to stand on the weighing platform to measure their accurate weight. In medical situations, it is widespread that patients attending the hospital will be in a lying position and hence brought on trolleys. These patients could not be made to stand, limiting the assessment of their body weight. In such situations, body weight is estimated by asking the patient about their latest recorded weight, viewing their previous medical records if available, asking the relatives for the patient's last recorded weight, and visual assessment, like does the patient “looks” thin or obese. Some objective methods include a calculation based on the formula using height, body circumference, gender, etc.

A study was done on 480 medical events due to failure in a medication drug dosage (8). The medication error resulted from a breakdown in obtaining, documenting, and communicating patients’ weight, resulting in under dosage or over dosage of prescribed medication. The authors concluded that the emergency department is most mentioned in the events, and a proper instrument is required to weigh the patients during admission to the hospital. A survey was carried out on the medication safety of patients prescribed common renally excreted drugs due to weight measurement failure (9). Weight-based dosing of renally excreted drugs like heparin, enoxaparin, and gentamicin are most commonly used in the general medical and surgical ward. In this study, a survey was carried out for three months. Patients are prescribed drugs like anticoagulants to treat venous thromboembolism and gentamicin to treat infection prophylaxis. Data were collected using interviews and questioning whether the patients were weighed or not. The study was used to characterize medication errors raised due to failure in weighing patients prescribed with therapeutic or prophylactic medication; the patients whose weighing is not done faced hemorrhage. The invention (10) uses an under-bed type weighing scale that fits over the bed frame and under the mattress. The dimensions of the scale are similar to that of a conventional bed used in hospitals; hence, it fits directly over the frame and does not require specially trained professionals to fit it over the bed. The scale consists of load cells located at the ends of a beam that runs along the transverse breadth of the scale. In another invention (11), the bed-type weighing scale consists of two frames: an upper measurement frame for supporting the patient on one side, attached with a load cell on the other at the middle, and a lower measurement frame connected to the rest of the stretcher body. A measurement unit calculates and displays the measured weight on the side of the bed. With an average error of 4.6 kg for an average weight of 72.9 kg, the prototype system created by Kim and Hong (12) estimates the body weight of an elderly patient resting on a smart mat in nonrestraint and unconscious situations. On the smart mat, 128 force sensing resistor (FSR) sensors were arranged in a 16 × 8 grid configuration. Pan et al. (13) made a study aimed at providing a self-designed indirect method to predict body weight through weight underneath the buttock (WUB) in the supine position.

Currently, available instruments for this purpose are bulky and use technologies that cannot be applied in all clinical settings (14, 15). Using a weighing bed is one of the best methods available to measure the body weight of individuals lying on the bed (16). There are many drawbacks to the existing weighing beds. These beds are not readily available for most hospitals; they are costly, not portable, and not user-friendly. In summary, it has been shown that weighing scales are not a useful tool for educating patients about their weight-bearing condition in the rehabilitation context. Even after receiving education and practicing on a set of scales, patients could not adhere to weight-bearing limits, according to a study (17). This result implies that other approaches for tracking weight-bearing levels under both static and dynamic circumstances should be investigated. A survey done among the health professionals involved in the weight assessment of patients indicates there is an urgent need to design a device that can be used to measure an individual's weight in a lying position. The device must be simple, portable, accurate, easy to calibrate, cost-efficient, and sensitive to measure the weight ranging between 500 g and 500 kg. Considering the above need, we aimed to design a trolley with load cell sensors that can measure an individual's body weight in a lying position. This device will be designed to be simple, portable, accurate, cost-effective, able to measure weight ranging from 500 g to 500 kg, display weight in both Kgs/Lb, battery operated, with auto-calibration and reset facility. This invention will help the medical personnel give the patient proper medication during emergency service, in the ICU, or during necessary surgery.

## 2 Methodology

**Figure 1:**
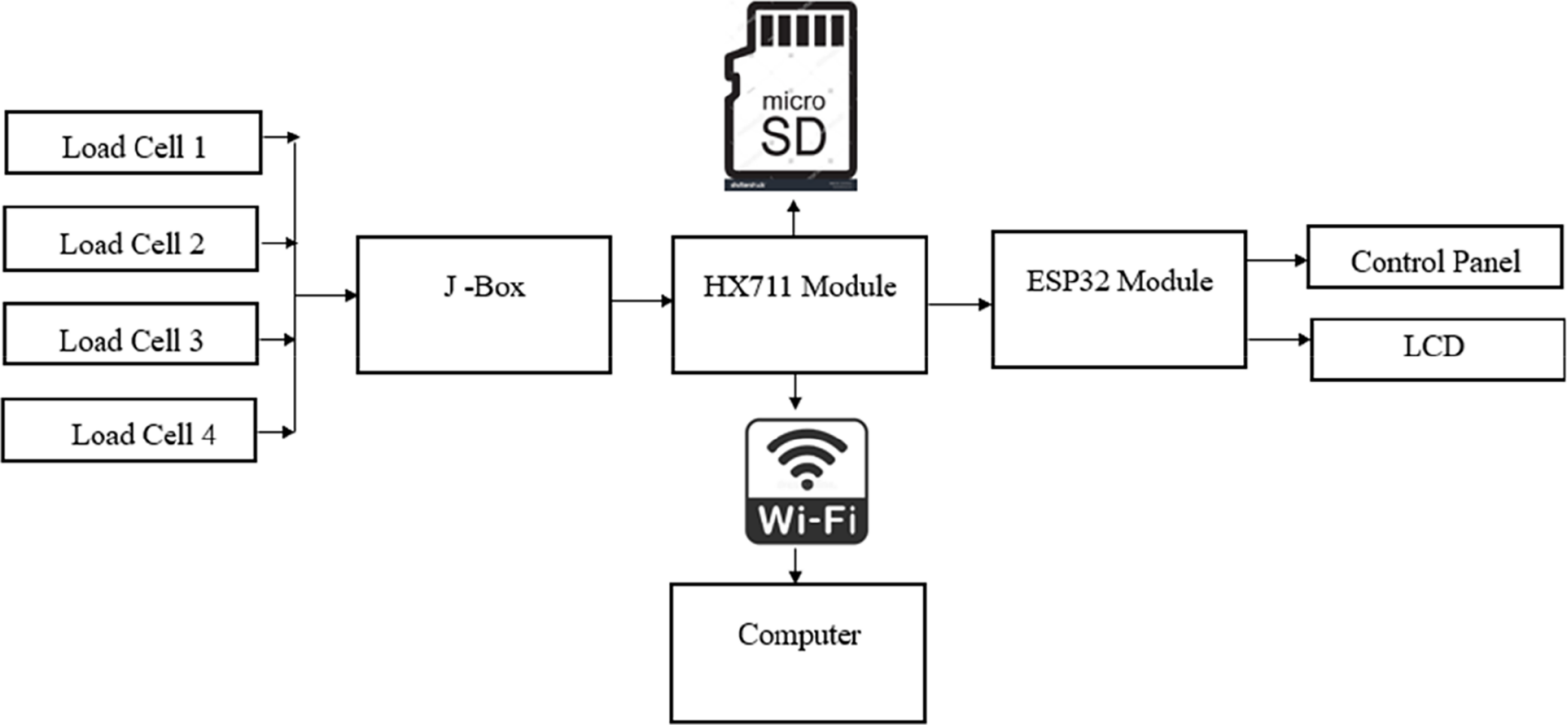
Hardware control panel.

This research aims to provide a method of weighing the patient in lying condition. The hardware control flow is shown in Figure 1. To achieve this, four strain gauge load cells are mounted beneath the upper part of the trolley where a patient will be placed. These load cells work on the principle of the Wheatstone Bridge. They produce a slight change in the output voltage when weight is applied to it. Since there are four load cells, there will be four different output voltages from each load cell. The need for four cells is to mount the cells on four corners of the trolley frame. This ensures the uniform weight distribution on the load cells to capture accurate weight. To maintain the stability of capturing the weight, the load cells are placed on four corners to avoid the influence of uneven movements while lying in the trauma position. A junction box signals the condition of these load cell outputs and has a single measurable output for the measuring device (i.e., Microcontroller). A junction box is an electronic circuit which sums and averages the multiple input coming from the load cells and provides a single output for the measuring device. The load cell output will be in the range of “mV”. It is difficult for the measuring instrument to detect this small voltage. The input should be amplified before it is passed to the measuring device. To amplify the input signal, the HX711 module is used. It has an amplification gain of 128 and a 24-bit precision ADC. The measuring device is ESP32, a ULP Microcontroller that comes with Bluetooth and Wi-Fi and can run on a clock speed of 240 MHz. The signal conditioning circuit comes with tare, reset, and save buttons for taking the weight and saving the current data to the database and arrow buttons (up and down) to move between the screens. A 16 × 2 LCD is used to display the weight measured and time of admission. The patient's weight will be displayed as soon as the patient is placed on the trolley. To avoid data loss, an external SD card is used to store data frequently for every 30 s, which can be accessed at any point in time. Data logging through Wi-Fi makes this invention feasible, which will help medical personnel access patient data from anywhere in the hospital. The electronic circuit and the load cells are powered by a battery, which can be recharged from the main power supply when the stretcher is free. A battery management system (BMS) is designed to provide a constant 5 V supply to the circuitry and charging circuit for the battery. The complete electronic circuits including the battery, were placed in the compartment under the stretcher.

### 2.1 Weighing scale functioning

**Figure 2:**
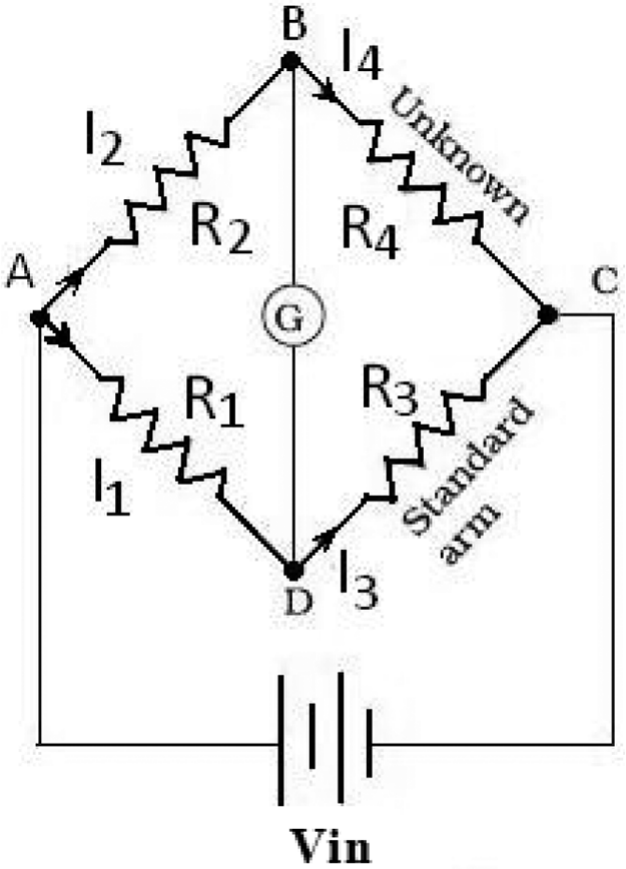
Wheatstone bridge.

Weight sensors are used to measure the weight of any object place on it. The load cell is a weight sensor, a transducer that converts the applied tension, compression, and pressure into a measurable electrical signal. This is commonly achieved through piezo-electric or strain gauges. Most load cell manufacturers use a strain gauge, an electric device that changes its resistance in response to load. These strain gauges are connected in the Wheatstone Bridge Circuit, as shown in Figure 2.

Four strain gauge sensors are connected as format shown above and provide regulated DC voltage excitation to the bridge circuit. The output voltage of the bridge circuit is in the range of millivolts per volt input, and this voltage should be amplified before sending the data to the Microcontroller.

(1) $$V_{\mathrm{out}}=\;V_{\mathrm{in}}\left(\frac{R_{3}}{R_{3}+\;R_{g}}-\;\frac{R_{2}}{R_{1}+\;R_{2}}\right)$$

The output voltage is calculated by using Equation 1. In the equation of Wheatstone Bridge, if R1, R2, R3, and R4 are equal, and a voltage Vin is applied between the terminal C and A, there will be zero potential difference between the output terminal D and B. When the value of R4 changes and it's not equal to R1, R2, and R3, the output terminal has some voltage difference.

## 3 Hardware design

The hardware design for this work includes electronic circuit design (microcontroller interface) and mechanical model rendering. The electronic design has complete circuit design, which combines interfacing different modules like LCD, SD card module, load cell amplifier to the microcontroller, and designing PCB for the entire signal conditioning circuit. The mechanical part includes creating a CAD model of the stretcher to be fabricated and designing the 3D printable case for the display panel and storage unit.

### 3.1 Electronic design

The electronic design is a combination of both hardware and software. On the hardware side, complete circuitry design is made, which includes selecting the appropriate electronic devices, interfacing different hardware parts to the microcontroller, and designing the schematic and PCB for the final signal conditioning circuit. The software side includes proper coding for the interfaces.

#### 3.1.1 ESP-32 micro-controller

**Figure 3:**
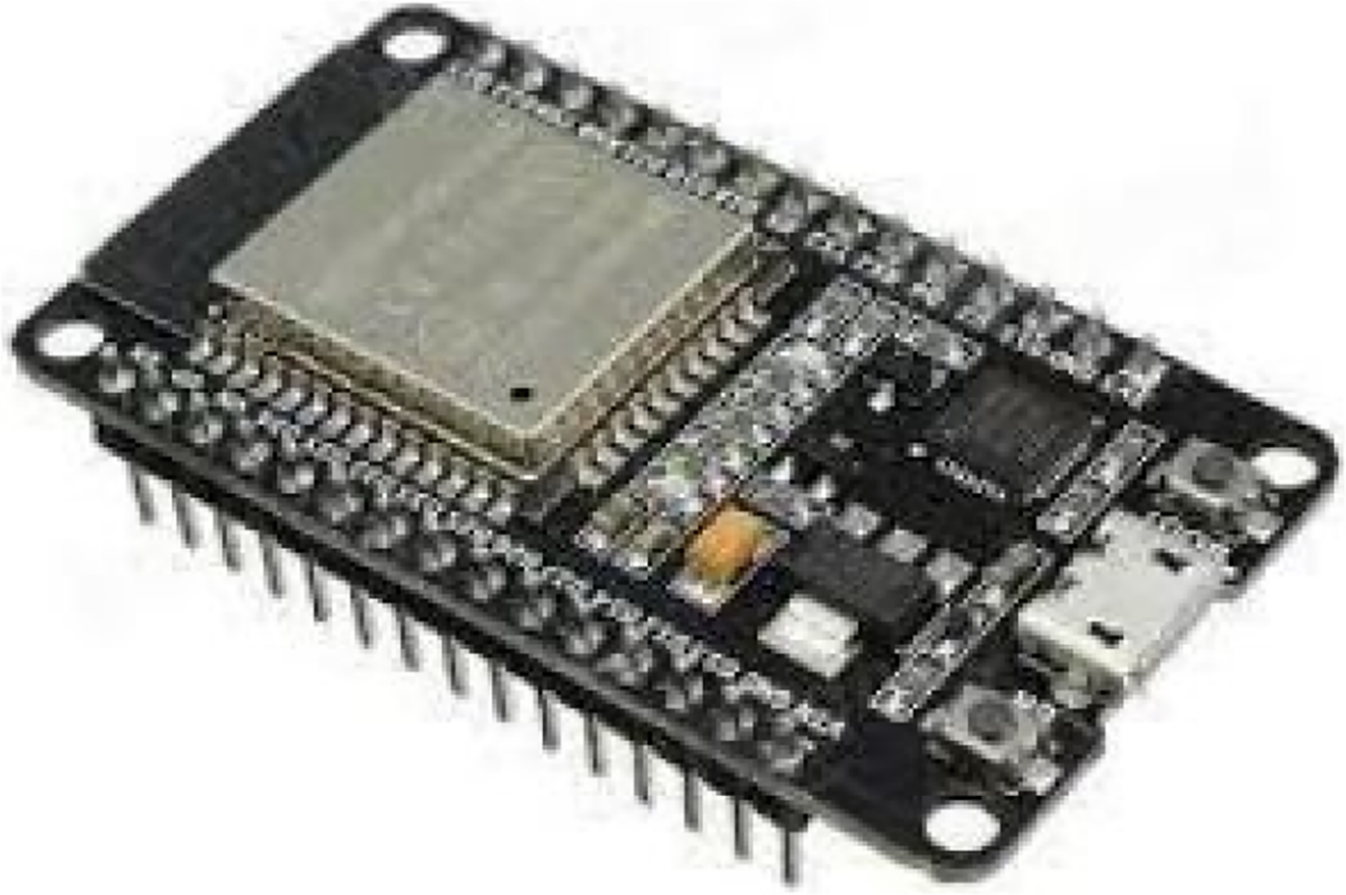
ESP-32 devKit V1.

ESP32 dev-kit V1, as shown in Figure 3, is a low-powered microcontroller designed by company Esspressif Systems, which runs on a 32-bit single/dual core CPU ESP32-D0WDQ6 chip platform. The MCU, built with Wi-Fi, BT, and BLE, makes the controller suitable for many IOT applications. The two CPU cores are independently tunable and operate at a frequency that may be adjusted between 80 and 240 MHz. The controller is appropriate for wireless applications due to its broad range and direct internet connectivity via a Wi-Fi router. ESP32, including high-speed SPI, UART, I2S, I2C, capacitive touch sensors, Hall sensors, SD card interface, Ethernet, and more integrate a wide variety of peripherals.

#### 3.1.2 Load cell

The load cell is a transducer that converts force such as tension, compression, pressure, or torque into electrical signals such as voltage or current. There are different load cell types like strain gauge, pneumatic hydraulic etc. This research selects a strain gauge load cell that uses a strain gauge sensor to measure the load applied. A strain gauge sensor is made up of very thin wire set up in a grid pattern that changes its electrical resistance when the shape of the strain gauge is altered.

#### 3.1.3 HX711 amplifier

HX711 is a precise 24-bit analog-to-digital converter (ADC) and amplifier intended for direct bridge sensor interfacing in industrial control applications and weigh scales. The low-noise programmable gain amplifier (PGA) receives a differential input from the input multiplexer that chooses either Channel A or B. Programming Channel A with a gain of 128 or 64 results in a full-scale differential input voltage of approximately ±20 mV or ±40 mV, respectively.

#### 3.1.4 Load cell junction box

A load cell junction box or summing box is an electronic device that combines multiple load cell input and give a single measurable output for the microcontroller. Summing box is used when more than one load cell is used in any system. It adds the signals from different load cells and gives a single signal representing the total load. All load cells should have the same capacity and outputs within 3.2% of each other.

#### 3.1.5 DS3231 RTC

**Figure 4:**
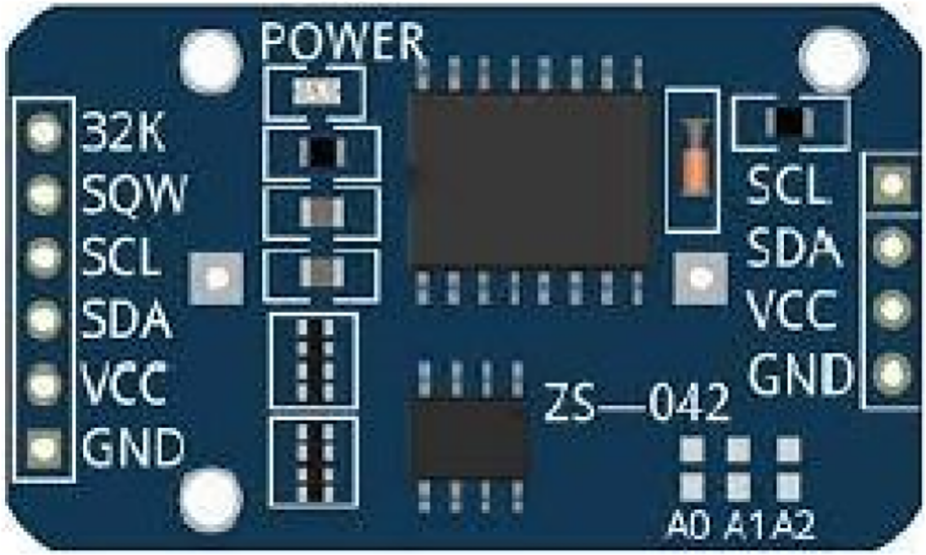
Ds3231 RTC.

The DS3231, as shown in Figure 4, is a low-cost, extremely accurate I^2^C real-time clock (RTC) with an integrated temperature-compensated crystal oscillator (TCXO) and crystal. The device incorporates a battery input and maintains accurate timekeeping when the main power to the device is interrupted.

### 3.2 Data logging over the air

The Internet of Things combines the network of physical objects, also called “things”, such as sensors, actuators, software, and other technologies together to connect and exchange data with multiple devices and systems over the Internet. The design includes data logging over the Wi-Fi every 30 s and displaying the data to the client i.e., to the hospital server database. To achieve this, NODE-RED is used, and with the help of MQTT, the data is transferred over the Wi-Fi to the NODE-RED. The data and the patient's demographic information are encrypted by the hospital's medical record-handling mechanism. Thus ensuring the data privacy and security of the patient.

#### 3.2.1 NODE-RED

**Figure 5:**
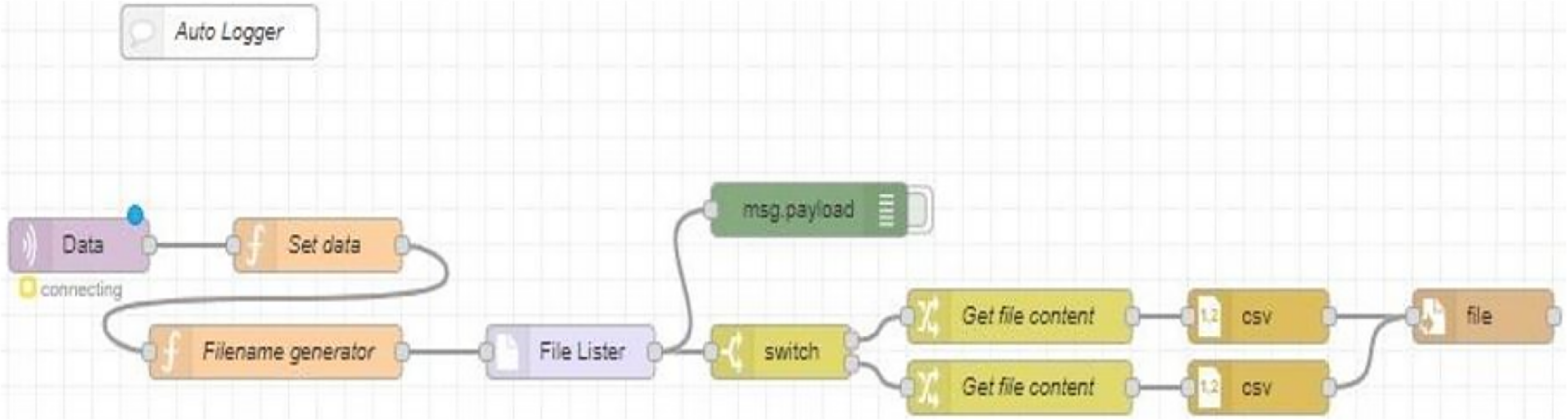
Node- Red flow for logging data to CSV file.

**Figure 6:**
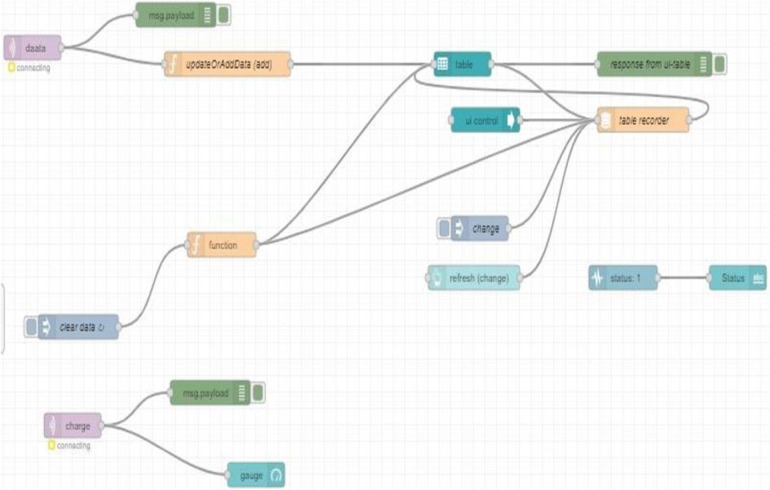
Node- Red flow for dashboard UI.

Node-RED is a cloud-based programming tool designed by IBM to bring together hardware devices, APIs, and online services as a part of the Internet of Things. It uses a browser-based flow editing tool to wire together the multiple nodes in the node palette, where all the nodes can be deployed together in a single click. It provides a visual observation for the user by designing a proper dashboard UI. As per the requirement, NODE-RED flow is designed such that a CSV file is generated every day, and it receives the data i.e., the weight and timestamp, and saves the data in the CSV file every 30 s for the whole day (Figure 5). A dashboard UI is created to monitor the saved data visually from a remote location. The data is transferred over the Wi-Fi using the MQTT protocol; node-red comes with built-in MQTT APIs making it more feasible for data logging (Figure 6).

#### 3.2.2 MQTT

The Message Queuing Telemetry Transport (MQTT) is a lightweight, Publish-Subscribe network protocol that transports messages between devices. It is designed for connection with remote locations where network bandwidth is limited. Messages can be published using a specified “Topic” and devices subscribed to the particular topic will receive the message every time it is published. For example, a weather station publishes a message on the topic “Air Speed” and clients who are subscribed to that topic receive the published message. The message is transported using the MQTT broker software running on the computer or a Raspberry PI, which functions as a host between the clients. The most commonly used MQTT broker is Mosquitto MQTT broker by the Eclipse, which has its APIs in the node-red palette.

### 3.3 Circuit schematic and PCB design

**Figure 7:**
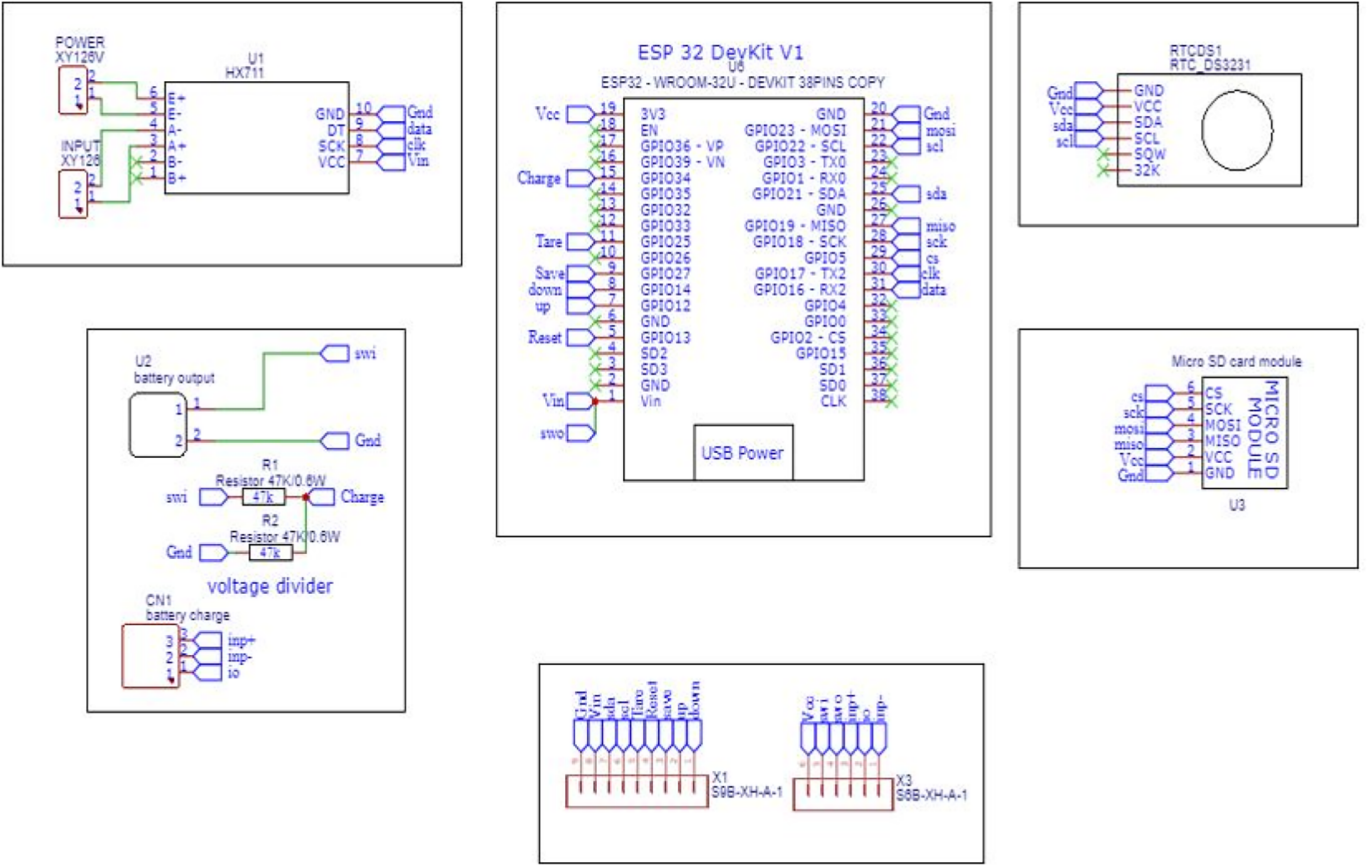
Main controller schematic.

**Figure 8:**
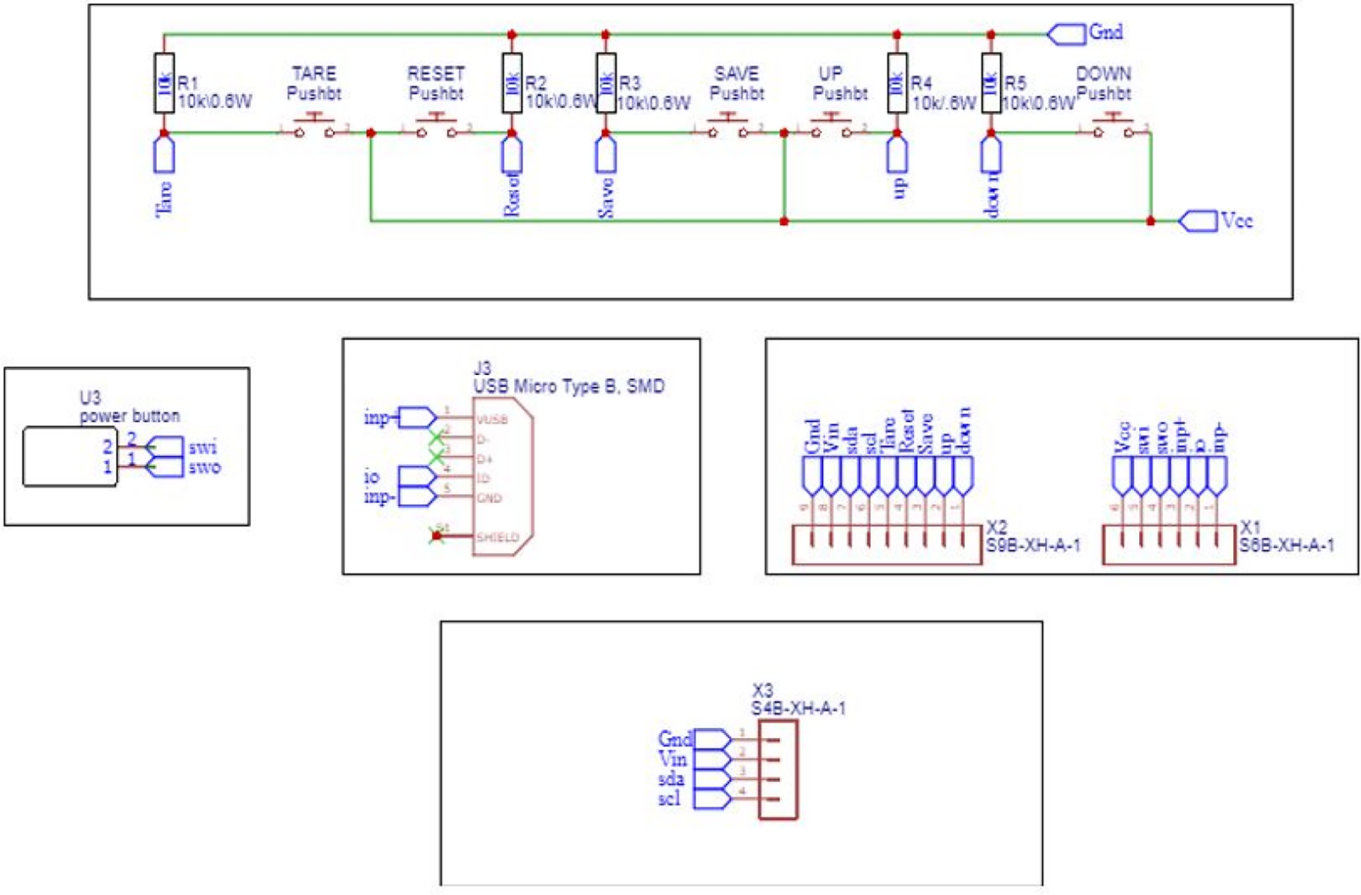
Display schematic.

**Figure 9:**
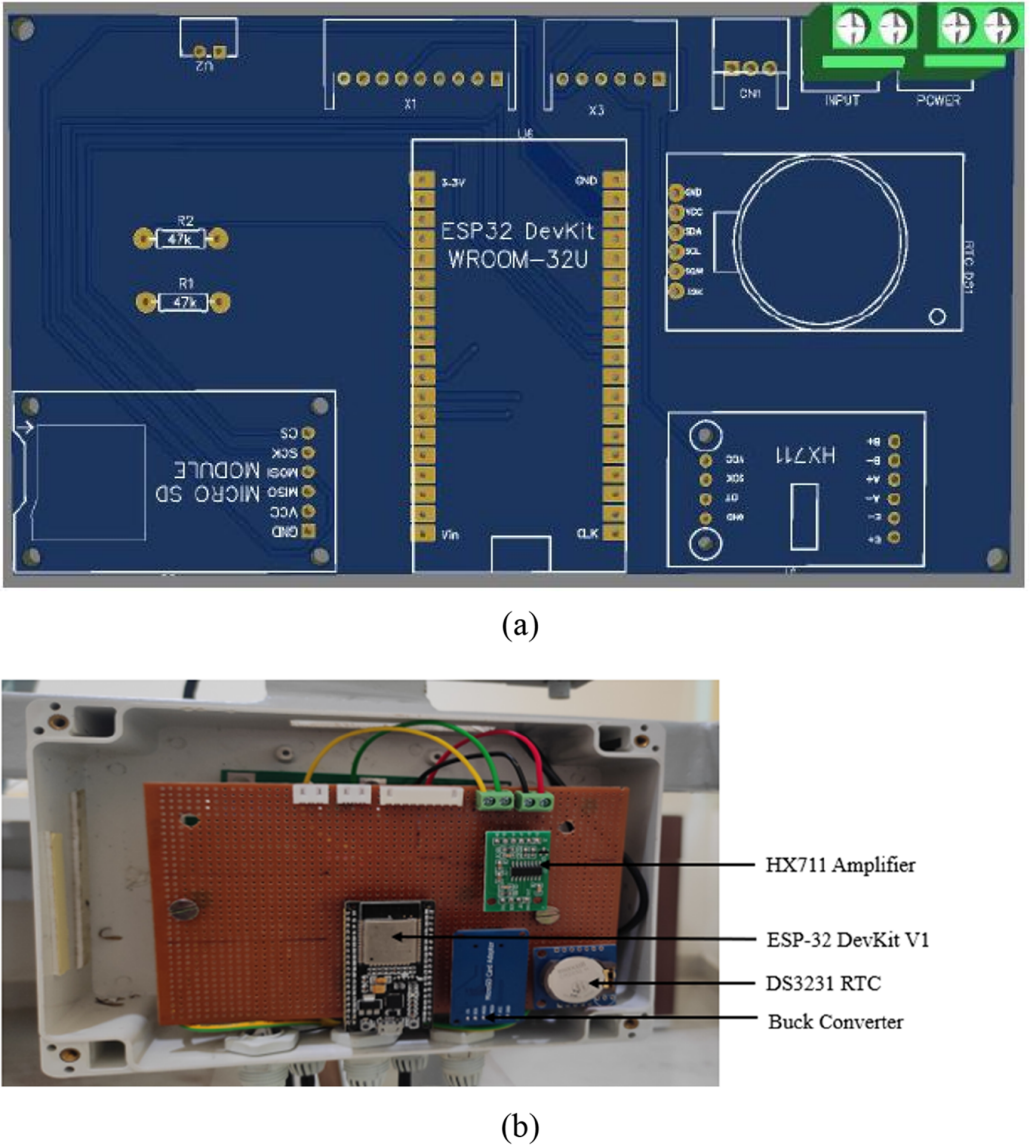
Main controller: **(A)** in printed circuit board **(B)** processor unit showing all components.

**Figure 10:**
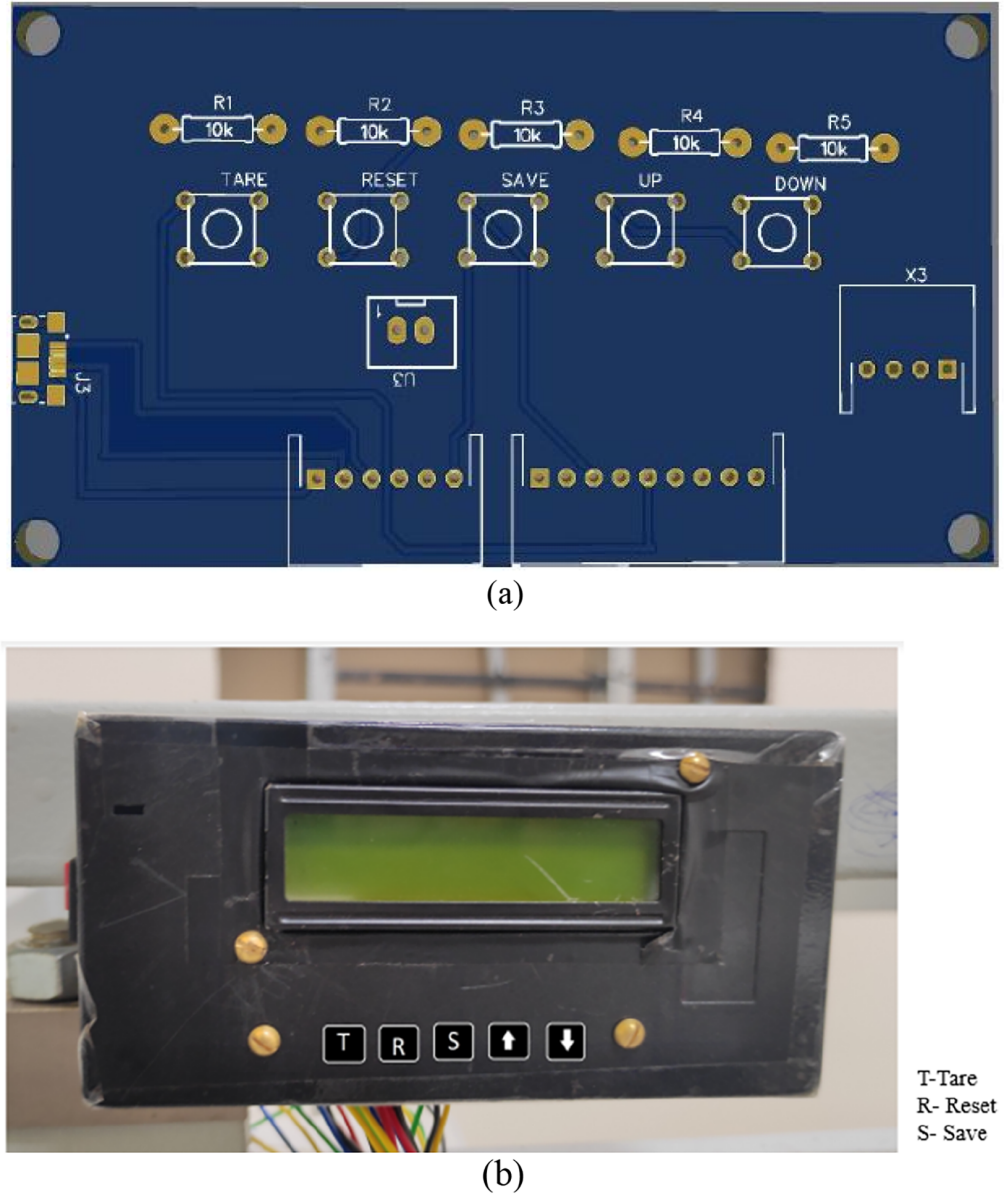
Display panel: **(A)** PCB **(B)** actual display unit.

The schematic design includes the interface of different modules like load cell, SD card module, RTC module, LCD to the microcontroller, and transforming the design into a PCB. The schematic of the Main controller and Display panel is shown in Figures 7, 8. The Schematic and PCB are designed using EasyEDA software. EasyEDA is a cloud-based online EDA design tool that enables the ability to design and simulate schematic, simulation, and printed circuit boards. As per the requirement of the work, two PCBs are designed: (i) Main Controller and (ii) Display panel. The main controller PCB includes a Controller, RTC, and load cell amplifier (Figure 9), and the display panel consists of the SD card module, LCD, and Micro-USB type B charging port (Figure 10).

### 3.4 CAD design

**Figure 11:**
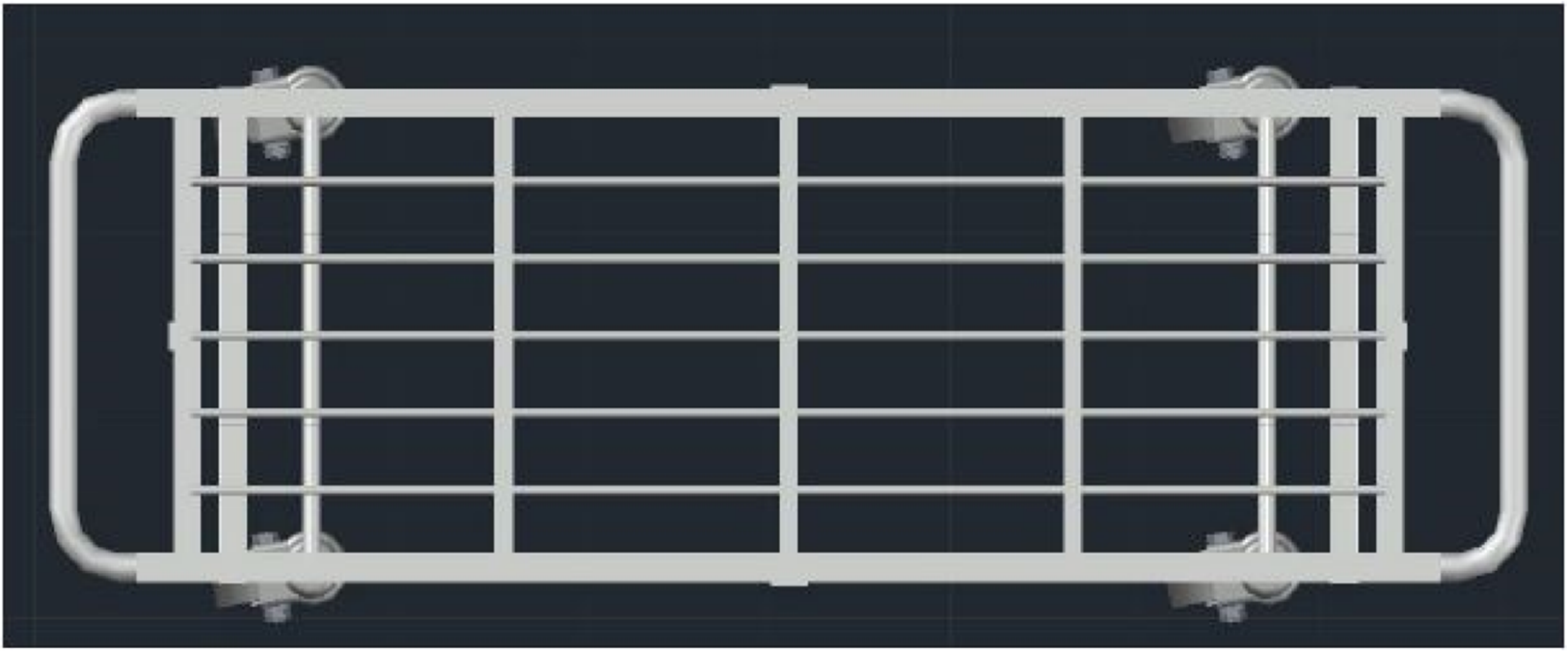
Top view of 3D model of the trolley.

**Figure 12:**
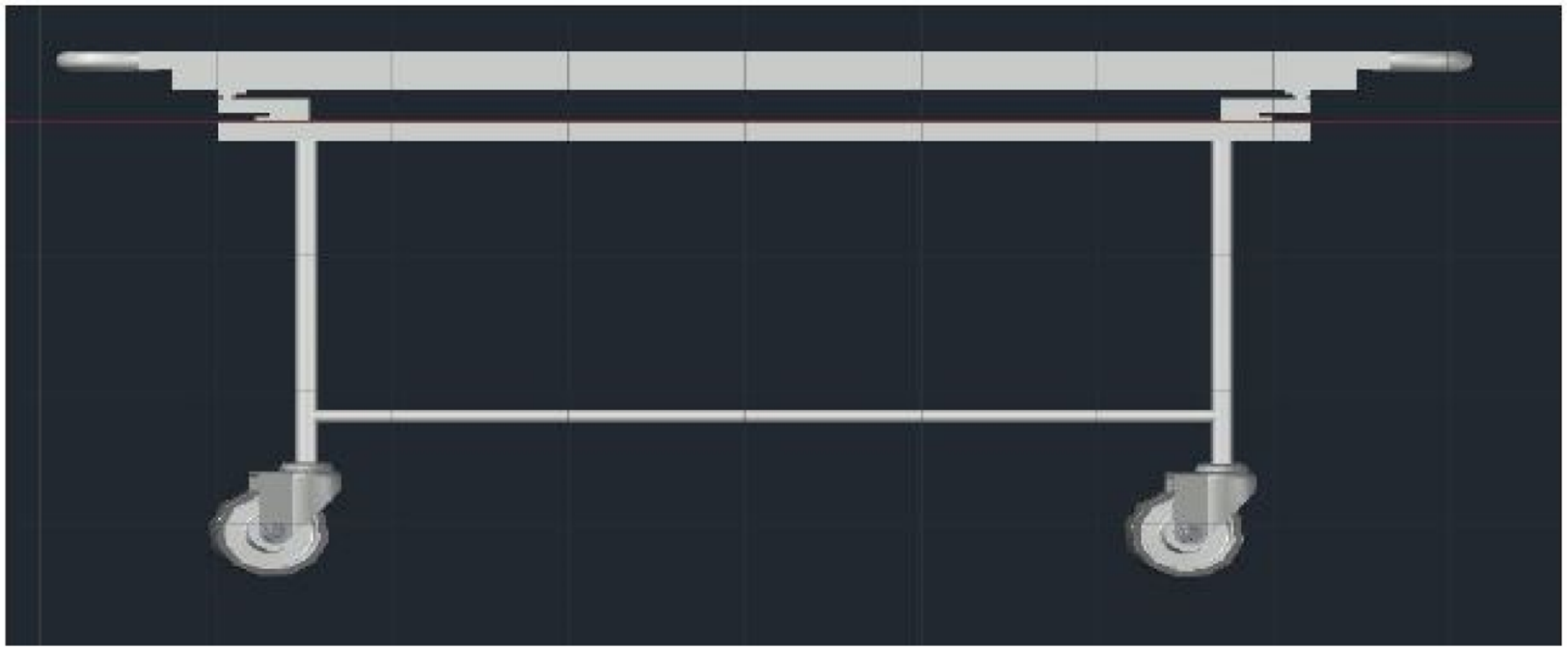
Side view of 3D model of the trolley.

The 3D model of the structure is designed using a CAD Tool, or computer-aided design and drafting (CADD), a technology for design and technical documentation that replaces manual drafting with an automated process. The complete mechanical structure uses AUTO-CAD, and the case is designed using the FUSION360 3D design tool. Figures 11, 12 show the top and side view of a 3D model of a custom-design Trolley.

### 3.5 Process flow of trolley usage

**Figure 13:**
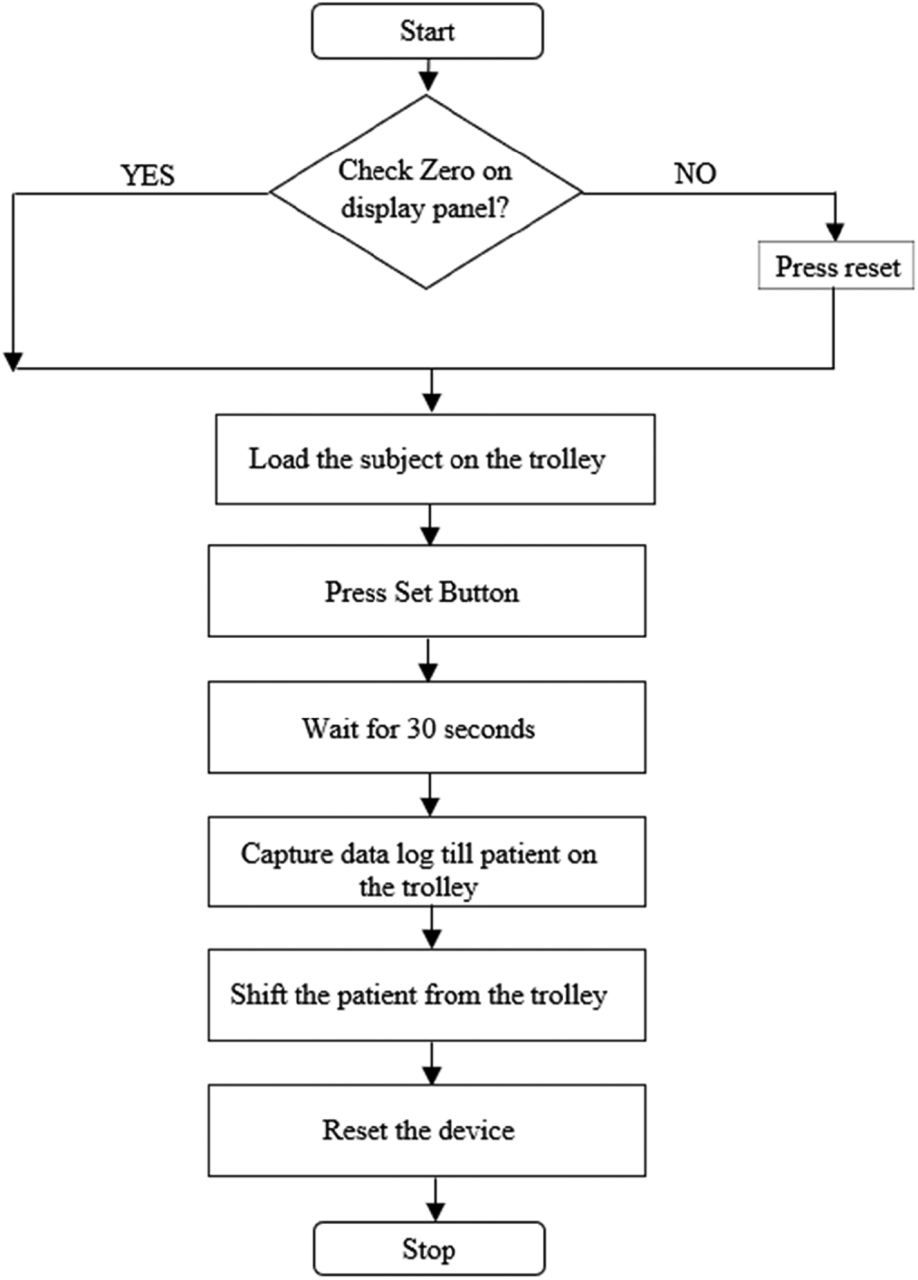
A flow chart of the trolley usage.

A special trolley has been designed with weight measuring sensors and display unit for this application. A microcontroller unit on the trolley performs data storage and display operations and will be connected to a nearby PC through wireless. Provisions have been made to auto data log for every 30 s on the remote PC. Communication has been tested for a range of 100 m. The display unit is designed with save, tare, reset buttons with complete battery backup and rechargeable option. The prototype has been tested with an accuracy of ±50 g with a full-scale range of 2–400 kg and the snapshots have been added here. A flow chart of the trolley usage is shown in Figure 13. Initially, the display panel made zero before placing the patient on the trolley. Load the subject on the trolley and press the set button. Wait for 30 s to get accurate weight reading and now, system continuously sends the weight information to the server. Once the measurement is completed, patient can be shifted from the trolley if required. Press reset button to halt the system.

## 4 Results and discussion

The code for the ESP32 interface is written in C language. Since the microcontroller supports Arduino IDE for programming, code compilation, debugging, and dumping are done using the same IDE.

### 4.1 Trolley fabrication

**Figure 14:**
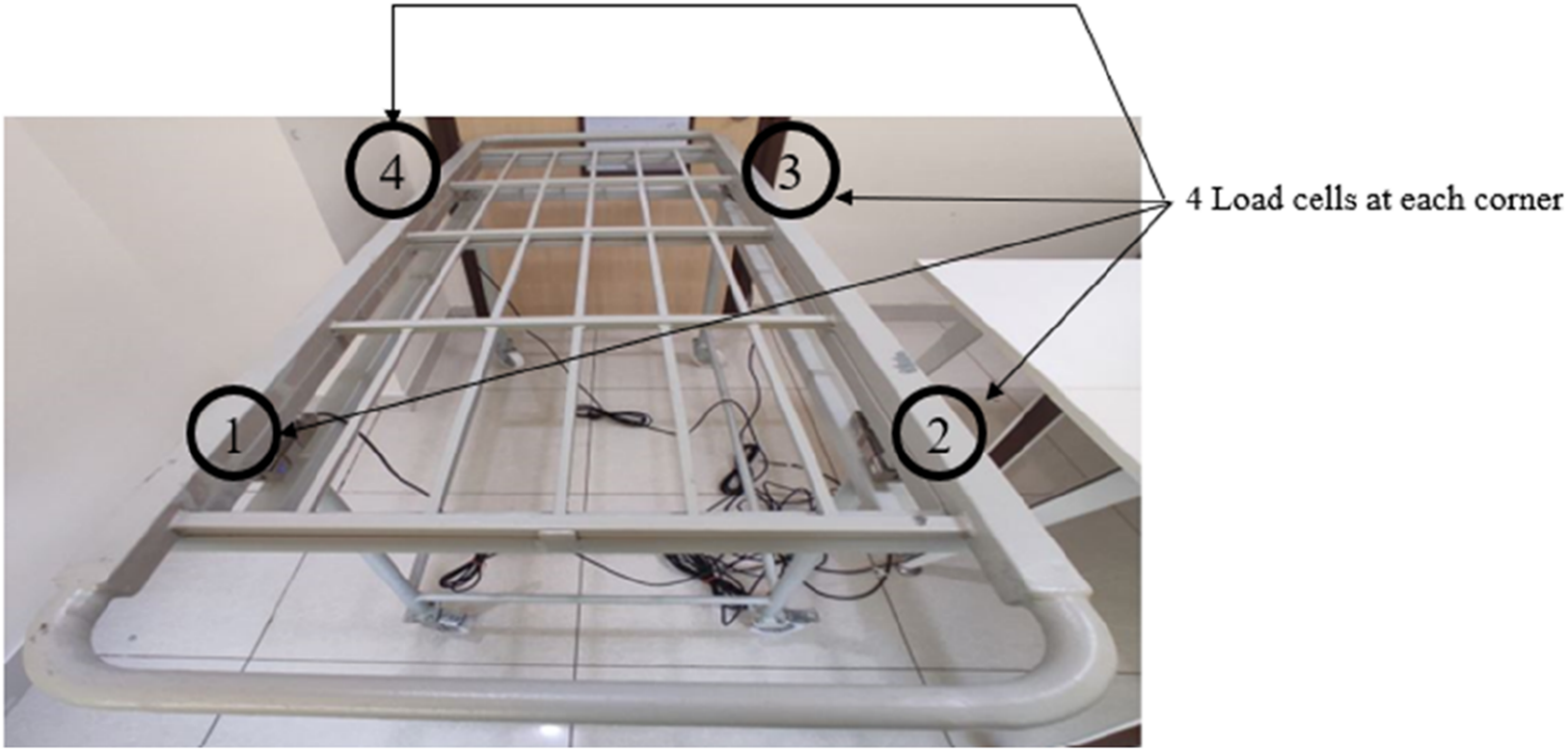
Fabricated trolley Top view.

**Figure 15:**
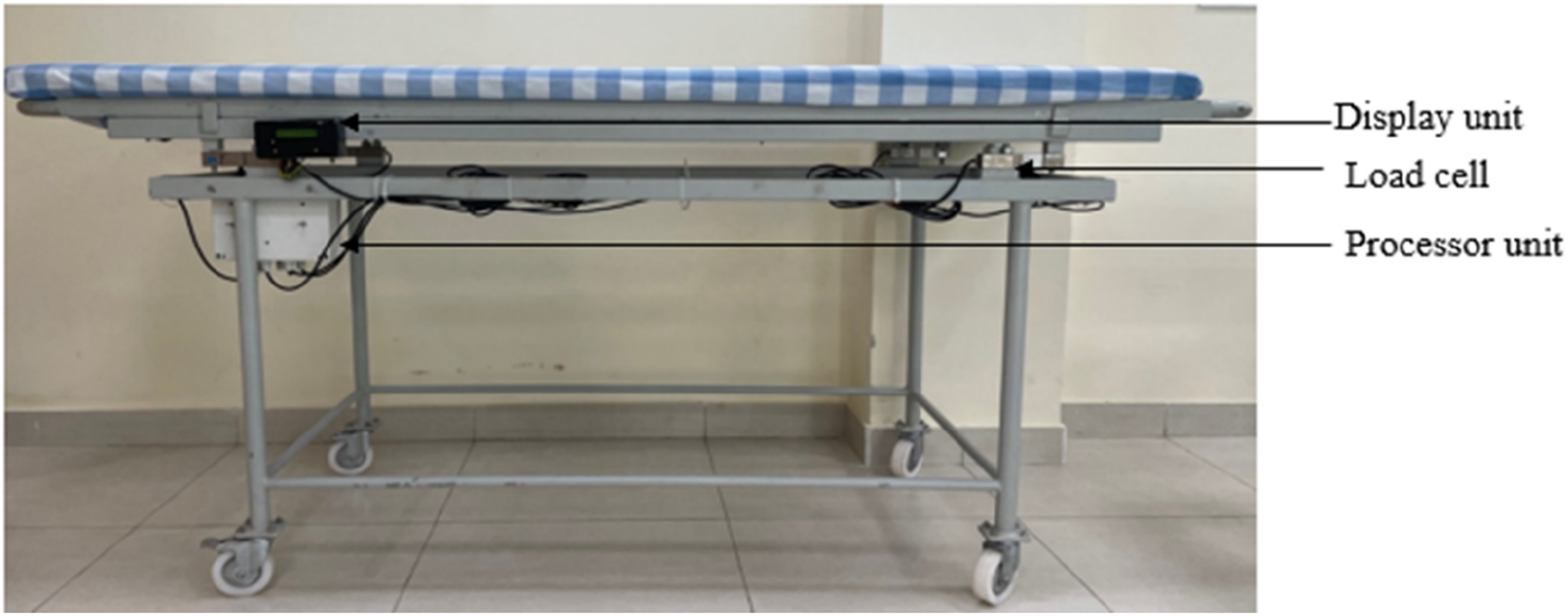
Fabricated trolley front view.

The motive is to design a prototype model of the trolley. The complete trolley is fabricated from local vendors and built using GI metal, which can withstand a good amount of load. It has a dimension of 75 × 24 × 28 inches (length × width × height) and is built into three parts 1. Top plate 2. Middle plate, and 3. Base plate. Figures 14, 15 show the fabricated trolley top and front view. The calibration mechanism of the sensor is added to the system itself. The initial position of the trolley is calculated. Set the button to zero reading then put the patient on the trolley and we get the weight of the patient in lying position. The regular servicing and sensor calibration schedule can be followed strictly as done for other medical instruments in the hospital.

### 4.2 Data monitoring and visualization

**Figure 16:**
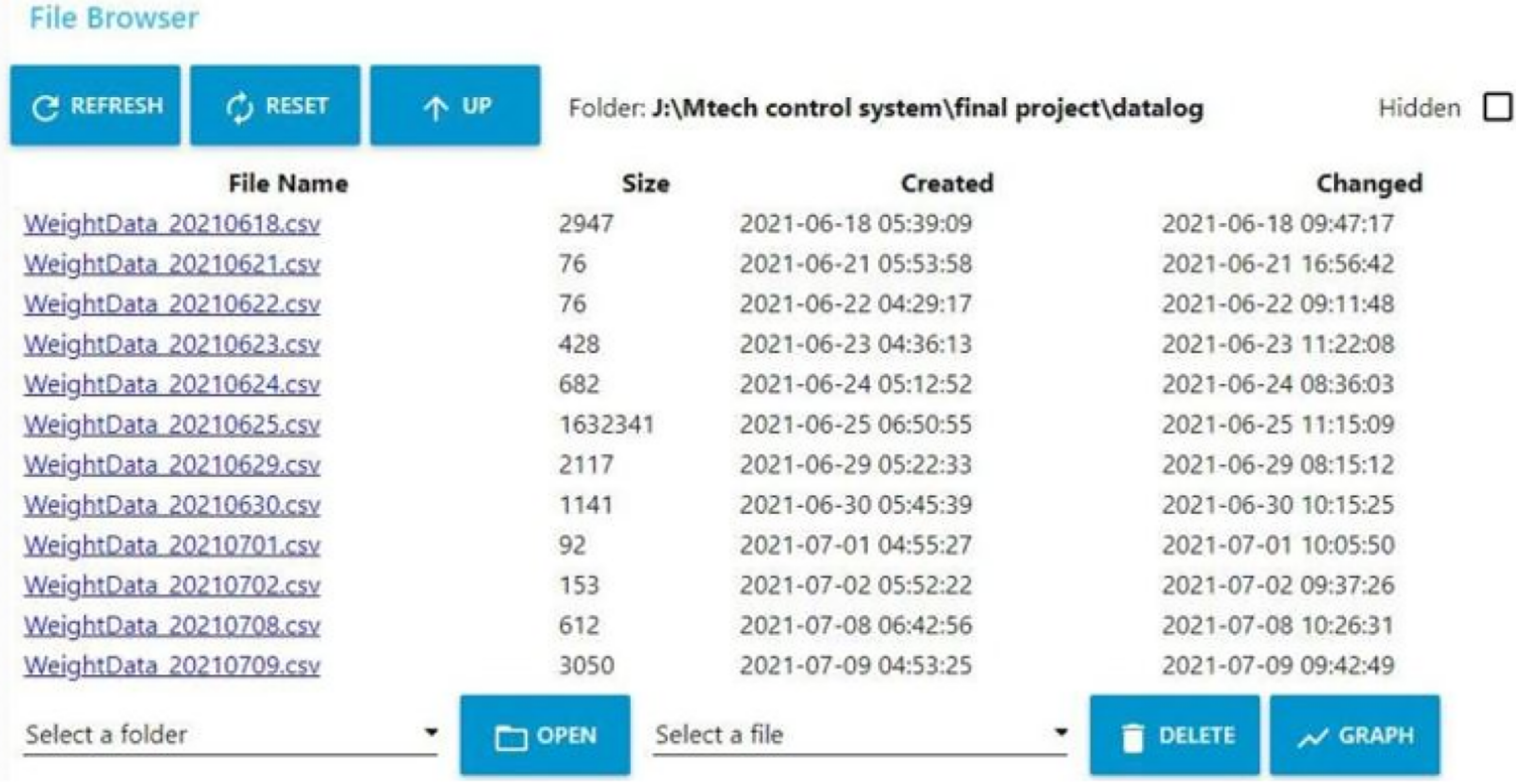
File browser user interface.

Medical personnel can visualize the patient weight from the side-mounted display panel and monitor the saved patient data log remotely using NODE- RED dashboard UI. The main UI in the dashboard consists of a table that keeps patient data and a gauge indicator to monitor the remaining battery power percentage. The Microcontroller sends data over the Wi-Fi and is saved as a CSV file; these files are saved locally on the device that hosts the NODE-RED server. Initially, the timestamp associated with the creation file is shown as soon as the patient is transferred to the trolly. The system captures the weight information periodically, and the CSV file is subsequently updated with the new weight values. The timestamp associated with each updation is shown on the dashboard's File-Browser User Interface (UI), as shown in Figure 15. The captured weight of the patient is sent to a centralized hospital server to store against the patient ID. It is assumed that the medical record handling system is taking care of privacy and confidentlity of the data. Files can be accessed through the File-Browser User Interface (UI) on the dashboard, as shown in Figure 16. The future work of this research is to build multiple stretchers embedded with weighing mechanisms, connected to a single server, and to save data in a single database. In addition, using a touch panel instead of adding multiple buttons provides feasible use. Making the system lighter in weight, making it easy for medical personnel to handle the stretcher. Limiting the number of loads to weigh the patient by single, double, or triple load cell structure cuts the overall structure price. Table 1: Comparative table highlighting features of the trolley method and other traditional methods used for weighing bedridden patients.

**Table 1:** Comparative table highlighting features of the trolley method and other traditional methods used for weighing bedridden patients.

Features	Trolley method	Traditional methods (e.g., bed scales, hoist scales)
Mobility	Without moving the patient's weight can be measured	Patients must bring onto the scale, causing discomfort
Ease of use	Simple setup and operation	Difficult with severe trauma patient (unconsciousness)
Safety	Minimal risk of patient injury during weighing	Minimum 2 caregivers support requires
Accuracy	Requires calibration one time before measurements	Well-calibrated, accuracy is good
Efficiency	Quick and convenient	Takes time and not efficient
Patient comfort	Reduce discomfort by avoiding movement	Often patients experience discomfort during the weighing process

### 4.3 Testing and validation

**Figure 17:**
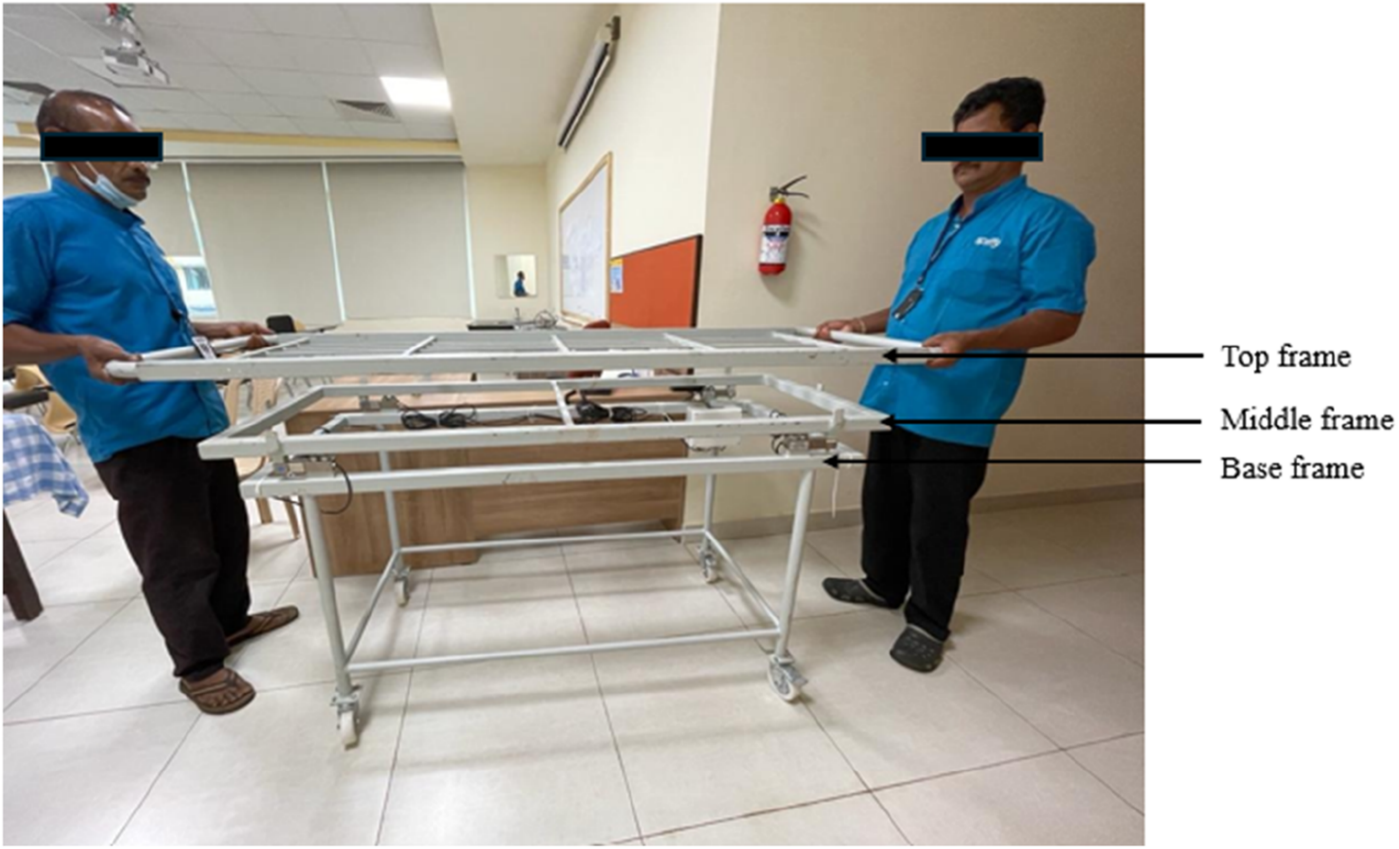
Designed trolley usage in hospital environment.

The designed trolley is the same as the hospital's existing trolley. Hence, there is no special requirement to consider the usability parameters. The weight-capturing apparatus is mounted on the trolley as an additional part as shown in Figure 17. A detachable top tray design has been adopted to make it easy while shifting the patient. The apparatus can be easily switched on with the button. Initially, the reset button will be pressed to calibrate the sensors and to initialize to zero before the patient lied on the bed. The set button will be pressed to capture the weight value. The procedure of weight capturing is provided to healthcare professionals. The usability aspect is validated by taking feedback on how fast the system captures the weight as soon as the button gets pressed. As per the feedback, the system starts capturing weight within 10 s. The regular servicing and sensor calibration schedule can be followed strictly for the designed trolley as is done for other medical instruments in the hospital.

In the validation and testing procedure, we captured the weight measurement of 10 healthy participants over 5 min using a designed trolley. Provision has been made to auto data log for every 30 s. Wireless communication has been tested for a range of 100 m, and a prototype has been tested with an accuracy of ±50 g and for a scale range of 2–140 kg. Once the participants’ average weight value is obtained from the developed trolley, it is compared against a traditional weighing scale (Table 2). The comparison of a person's weight between a traditional weighing scale and a designed trolley assesses how closely the new trolley system aligns with the gold standards. Interestingly, slight variation between the developed trolley and the traditional scale is displayed for a same participant. Weighing in the lying condition is slightly less (∼50 g) when compared with the traditional scale. This is because variations in gravitational force in lying conditions marginally influence weight measurement.

**Table 2:** Weight comparision of the participants between average system weight vs. weighing scale.

Patient ID (years)	Average system weight (kg) (New trolley design)	Weighing scale weight (kg) (Traditional method)
1 (2.5 years)	10.2	10.6
2 (17 years)	47.8	48
3 (20 years)	52.4	52.8
4 (25 years)	64	64.4
5 (30 years)	68	68.3
6 (36 years)	54.6	55
7 (42 years)	62	62.1
8 (55 years)	72	72.4
9 (72 years)	59.5	60
10 (81 years)	138	138.2

## 5 Conclusion

Weighing the patient's weight in lying condition is a common practice in rehabilitation settings to assess their weight-bearing status. Recording patient data like height and weight is an integral part of nursing practice. By regularly measuring and tracking the patient's weight in a lying condition, healthcare professionals can gather important data on fluid overload levels and evaluate the effectiveness of diuresis therapy. This information can help determine the patient's response to treatment and guide further interventions. Furthermore, weighing the patient's weight in a lying condition provides valuable insights into their overall health status, such as identifying any fluctuations in body fluid volume and detecting signs of fluid overload or dehydration. This research work aims to provide a total solution for weighing patients in lying condition by designing a trolley, which can carry the patient and measure the weight of the patient on it.

## Data Availability

The original contributions presented in the study are included in the article/Supplementary Material, further inquiries can be directed to the corresponding author.
